# OPA1 overexpression ameliorates mitochondrial cristae remodeling, mitochondrial dysfunction, and neuronal apoptosis in prion diseases

**DOI:** 10.1038/s41419-019-1953-y

**Published:** 2019-09-24

**Authors:** Wei Wu, Deming Zhao, Syed Zahid Ali Shah, Xixi Zhang, Mengyu Lai, Dongming Yang, Xiaoqian Wu, Zhiling Guan, Jie Li, Huafen Zhao, Wen Li, Hongli Gao, Xiangmei Zhou, Jian Qiao, Lifeng Yang

**Affiliations:** 1 Key Laboratory of Animal Epidemiology and Zoonosis, Ministry of Agriculture, National Animal Transmissible Spongiform Encephalopathy Laboratory, College of Veterinary Medicine, China Agricultural University, Beijing, China; 2 Department of Pathophysiology, College of Veterinary Medicine, China Agricultural University, Beijing, China; 3 Department of Pathology, Faculty of Veterinary Sciences, Cholistan University of Veterinary and Animal Sciences, Bahawalpur, Pakistan

**Keywords:** Apoptosis, Cell death in the nervous system

## Abstract

Prion diseases caused by the cellular prion protein (PrP^C^) conversion into a misfolded isoform (PrP^Sc^) are associated with multiple mitochondrial damages. We previously reported mitochondrial dynamic abnormalities and cell death in prion diseases via modulation of a variety of factors. Optic atrophy 1 (OPA1) is one of the factors that control mitochondrial fusion, mitochondrial DNA (mtDNA) maintenance, bioenergetics, and cristae integrity. In this study, we observed downregulation of OPA1 in prion disease models in vitro and in vivo, mitochondria structure damage and dysfunction, loss of mtDNA, and neuronal apoptosis. Similar mitochondria findings were seen in OPA1-silenced un-infected primary neurons. Overexpression of OPA1 not only alleviated prion-induced mitochondrial network fragmentation and mtDNA loss, decrease in intracellular ATP, increase in ADP/ATP ratio, and decrease in mitochondrial membrane potential but also protected neurons from apoptosis by suppressing the release of cytochrome c from mitochondria to cytosol and activation of the apoptotic factor, caspase 3. Our results demonstrated that overexpression of OPA1 alleviates prion-associated mitochondrial network fragmentation and cristae remodeling, mitochondrial dysfunction, mtDNA depletion, and neuronal apoptosis, suggesting that OPA1 may be a novel and effective therapeutic target for prion diseases.

## Introduction

Prion diseases are transmissible fatal neurodegenerative disorders, which occur in humans (e.g., Creutzfeldt–Jakob Disease [CJD], Kuru, and so on) and other mammalian species (bovine spongiform encephalopathy [BSE] in cattle, scrapie in sheep, chronic wasting disease [CWD] in cervidae)^1^. Prion diseases are caused by accumulation of protease-resistant misfolded form (PrP^Sc^) rich in beta-sheet structure of cellular prion protein (PrP^C^), mainly consisting of alpha-helical structure^2,3^. Typical lesions of prion diseases are spongiform vacuolation, proliferation of astrocytes, and neuronal loss in the central nervous system (CNS)^3,4^. The neurotoxic PrP fragment 106–126 (PrP^106–126^) has similar chemical and pathogenic properties to PrP^Sc^, including high beta-sheet structure and proteinase K resistance, and causes neuronal apoptosis and induces proliferation of astrocytes. Consequently, PrP^106–126^ is used as a model peptide of PrP^Sc^ for the study of prion diseases^5–7^.

Mitochondrial damage and dysfunction is a common feature in prion diseases and other neurodegenerative conditions. Abnormalities of mitochondrial dynamics occur in the early stage of neurodegenerative diseases^8^. The mitochondrion is a dynamic organelle and constantly divides and fuses to maintain sufficient energy to the cell, which is critical to cell viability and function^9,10^. The protein machinery that controls mitochondrial dynamics is a set of large dynamin-related GTPase proteins. The dynamin-like protein 1 (DLP1) is a key player in mitochondria fission process; during fission, cytosolic DLP1 translocates to mitochondrial outer membrane by receptor proteins, such as fission 1 (Fis 1) and mitochondria fission factor (Mff)^11–13^. The mitofusins 1/2 (MNF1 and MFN2) are required for fusion of the outer mitochondrial membrane; MFN2 is considered to play a major regulatory role and MFN1 tethers two juxtaposed organelle^14,15^. Optic atrophy 1 (OPA1) localizes to the inner mitochondrial membrane (IMM) and, together with MFN1, induces mitochondrial fusion^16^, maintains mitochondrial DNA (mtDNA) replication and distribution and also stabilizes bioenergetics efficiency^17,18^. In addition, OPA1 also controls apoptosis through cristae remodeling and cytochrome c release independently from mitochondrial fusion^19,20^.

In a previous study, we reported mitochondrial fragmentation and dysfunction in prion diseases, and inhibition of DLP1 markedly ameliorates PrP^106–126^-induced mitochondrial dysfunction and neuronal death. In this study, we investigated the role of OPA1 in prion-induced abnormalities of mitochondrial dynamics and neuronal apoptosis. Our research shows that the protein level of OPA1 is dramatically downregulated in in vitro and in vivo models of prion diseases, and OPA1 overexpression alleviated PrP^106–126^-induced mitochondrial cristae collapse, mtDNA depletion, mitochondrial dysfunction, and neuronal apoptosis. Our results indicate that OPA1 may be another potential therapeutic target of prion diseases.

## Materials and methods

### Animal ethics approval

All animal experiments were conducted according to the guidelines of Beijing Municipality on the Review of Welfare and Ethics of Laboratory Animals and approved by the Beijing Municipality Administration Office of Laboratory Animals.

### Mouse prion diseases model

Female C57Bl/6 mice aged 4–6 weeks (*n* = 20) were randomly divided into two groups. One group (*n* = 10) was inoculated intracerebrally with the RML scrapie agent, and the other group (*n* = 10) was used as age-matched controls. The mice were sacrificed when the animals developed late-stage prion disease based on clinical signs.

### Primary cortical neuron culture

Cerebral cortex neurons were isolated from a 1-day-old neonatal rat as described previously^21^. Briefly, cells were dissociated after digestion with Dulbecco’s modified Eagle’s medium (DMEM) containing 2 mg/mL papain (Invitrogen, Carlsbad, CA, USA) and 50 mg/mL DNAse (Sigma-Aldrich, St. Louis, MO, USA) for 30 min at 37 °C. The dissociated cells were plated at a density of 5 × 10^5^cells per well and cultured in Neurobasal-A Medium (Invitrogen, USA) containing 2% serum-free B27 supplement (Invitrogen, USA) and 0.5% Penicillin–Streptomycin (Gibco, USA). After 2 days, 10 μM cytarabine (Sigma, USA) were added to control glial cell growth. Then half of the medium was replaced by fresh medium every 2 days. Experimental treatments were performed after culture for 7 days.

### Plasmids and transfection

OPA1 small interfering RNA (siRNA; sequence: 5′-GUUAUCAGUCUGAGCCAGGTT-3′) was obtained from Synbio Technologies, Suzhou. The pCAG-OPA1 plasmid was obtained from Vitalstar Biotechnology, Beijing. Primary neurons were transfected with siRNA or pCAG-OPA1 plasmid using the Lipofectamine 3000 reagent (Invitrogen, USA) in Opti-MEM (Invitrogen, USA) for 48 h following the manufacturer’s instructions.

### Prion protein peptide

PrP peptide PrP^106–126^ (sequence: KTNMKHMAGAAAAGAVVGGLG) of 98% purity was synthesized by Sangon Bio-Tech (Beijing, China). The peptide was dissolved in 0.1 M phosphate-buffered saline (PBS) to a concentration of 1 mM and allowed to aggregate at 4 °C for 24 h. Experiments were conducted with the final peptide concentration of 150 μM.

### Mitochondrial and cytoplasmic protein isolation

Mitochondrial and cytoplasmic proteins were extracted from brain tissue or neuron cell culture using a Qproteome Mitochondria Isolation Kit (Qiagen, Germany).

Cells or freshly excised tissues were homogenized with a lysis buffer (Qiagen, Germany) supplemented with a Protease Inhibitor (Qiagen, Germany) and centrifuged at 1000 × *g* for 10 min at 4 °C. The supernatant contains mainly cytosolic proteins and was frozen for western blotting for cytosolic cytochrome c. The pellet was resuspended in an ice-cold disruption buffer (Qiagen, Germany) and centrifuged at 1000 × *g* for 10 min at 4 °C. The supernatant was transferred to a 1.5-ml tube and centrifuged at 6000 × *g* for 10 min at 4 °C. The resulting mitochondrial pellet was washed with a mitochondria storage buffer (Qiagen, Germany) and centrifuged at 6000 × *g* for 20 min at 4 °C. The mitochondrial pellet was resuspended in RIPA lysis buffer (Beyotime, China) and then frozen for western blotting.

### Western blotting

Specific proteins expressed in the mouse brain and neuron cell cultures were determined by western blotting using specific antibodies. Proteins (40 µg) were separated on 10% or 12% sodium dodecyl sulfate-polyacrylamide gel electrophoresis gels and then transferred onto a nitrocellulose membrane. Nonspecific binding sites were blocked using 5% fat-free dried milk in 1 × TBST at 37 °C for 1 h. The membrane was incubated at 4 °C overnight with individual primary antibodies, including rabbit anti-OPA1 (Cell Signaling Technology, USA), rabbit anti-MFN1 (Proteintech, USA), mouse anti-MFN2 (Abcam, USA), rabbit anti-cytochrome c (Cell Signaling Technology, USA), rabbit anti-cleaved caspase 3 (Cell Signaling Technology, USA), mouse anti-GAPDH (Proteintech, USA), rabbit anti-β-actin (Proteintech, USA), and rabbit anti-VDAC (Cell Signaling Technology, USA). After washing with TBST, the membrane was incubated with secondary antibodies, goat anti-mouse IgG or anti-rabbit IgG conjugated to horseradish peroxidase (1:10,000). The blots were visualized using a Chemiluminescent Imaging System (Tanon Science and Technology).

### mtDNA content

Total DNA was extracted from tissue or cultured cells and quantified by spectrophotometry (NanoDrop 2000). Real-time PCR was performed using the ViiA7 Fast Real-Time PCR System (ABI) and SYBR Green Master Mix (Bio-Rad). Primer sequences were as follows: mtDNA (F:5′-CCTATCACCCTTGCCATCAT-3′ and R:5′-GAGGCTGTTGCTTGTGTGAC-3′) and nuclear DNA (nDNA) (F:5′-ATGGAAAGCCTGCCATCATG-3′ and R:5′-TCCTTGTTGTTCAGCATCAC-3′). Quantification was expressed as fold change relative to control samples calculated by the comparative CT method (2^−ΔΔCT^).

### Measurement of ATP levels, ADP/ATP ratio, and mitochondrial transmembrane potential

ATP levels and ADP/ATP ratio in cultured cells were evaluated using an ATP Assay Kit (Beyotime, China) and ADP/ATP Ratio Bioluminescence Assay Kit (Biovision, USA), respectively, according to the manufacturer’s instructions. Luminescence was measured using a GloMax® 96 Microplate Luminometer. Mitochondrial transmembrane potential was measured using the Mitochondrial Membrane Potential Assay Kit with JC-1 (Beyotime, China). Primary neurons were incubated with DMEM medium containing 10 μM JC-1 at 37 °C for 20 min and then washed twice with PBS. Fluorescence signals were analyzed by a BD fluorescence-activated cell sorter Calibur.

### Immunohistochemistry (IHC)

OPA1 in brain tissue was visualized by IHC. Brain tissue was fixed in 10% buffered formalin solution and embedded in paraffin. Sections of about 5-μm thick were stained with the anti-OPA1 antibody (Abcam, USA) using the protocol described previously by Yang et al.^22^. Digital images were analyzed under an Olympus DP72 microscope.

### Immunofluorescence microscopy

Primary neuronal cells were seeded on 24-well cover slips and then transfected with Mito-GFP (Clontech, USA) before treatment with PrP^106–126^ or control peptide. Fluorescence images of mitochondria were acquired using an Olympus confocal microscope. Measurement of mitochondrial length was performed using Image J (National Institutes of Health, USA).

### Transmission electron microscopy

Brain samples of approximately 2 × 2 × 2 mm^3^ in size and pellets of cultured primary neuron cells were fixed in ice-cold 5% glutaraldehyde in 0.1 M sodium cacodylate buffer (pH 7.4) at 4 °C. The tissue or cells fixed in gluraraldehyde were rinsed with sodium cacodylate buffer and then further fixed in 1% OsO_4_ in 0.1 M sodium cacodylate buffer on ice for 2 h before dehydration with acetone. The tissue and cell pellets were embedded in resin before polymerization at 60 °C for 48 h. Ultrathin sections (70 nm) were mounted onto copper grids and counterstained with 4% uranyl acetate and lead citrate before observation under a transmission electron microscope (JEM-1230, Japan) operating at 80 kV.

### Cell viability assay

Viability of neuron cells in response to peptide treatment was evaluated using the Cell Counting Kit-8 (CCK-8) assay (Beyotime, China). Briefly, the CCK-8 solution was added to primary cultures of rat cortical neurons and incubated for 1 h at 37 °C and 5% CO_2_. The absorbance of the samples was obtained at 450 nm using a microplate reader (ThermoFisher, USA). The cell viability of treated cultures was expressed as the percentage of the control culture.

### Statistical analysis

All assays were repeated at least three times. Data were expressed as means ± SEM. All comparisons for parametric data were analyzed using Student’s *t* test or one-way analysis of variance followed by post hoc Tukey’s test using the SPSS software (version 13.0: SPSS Inc., Chicago, IL, USA), GraphPad Prism 5 software (La Jolla, CA, USA), and Image J (National Institutes of Health, USA). *P* < 0.05 was considered statistically significant.

## Results

### OPA1 is downregulated in prion disease models in vivo and in vitro

**Fig. 1:**
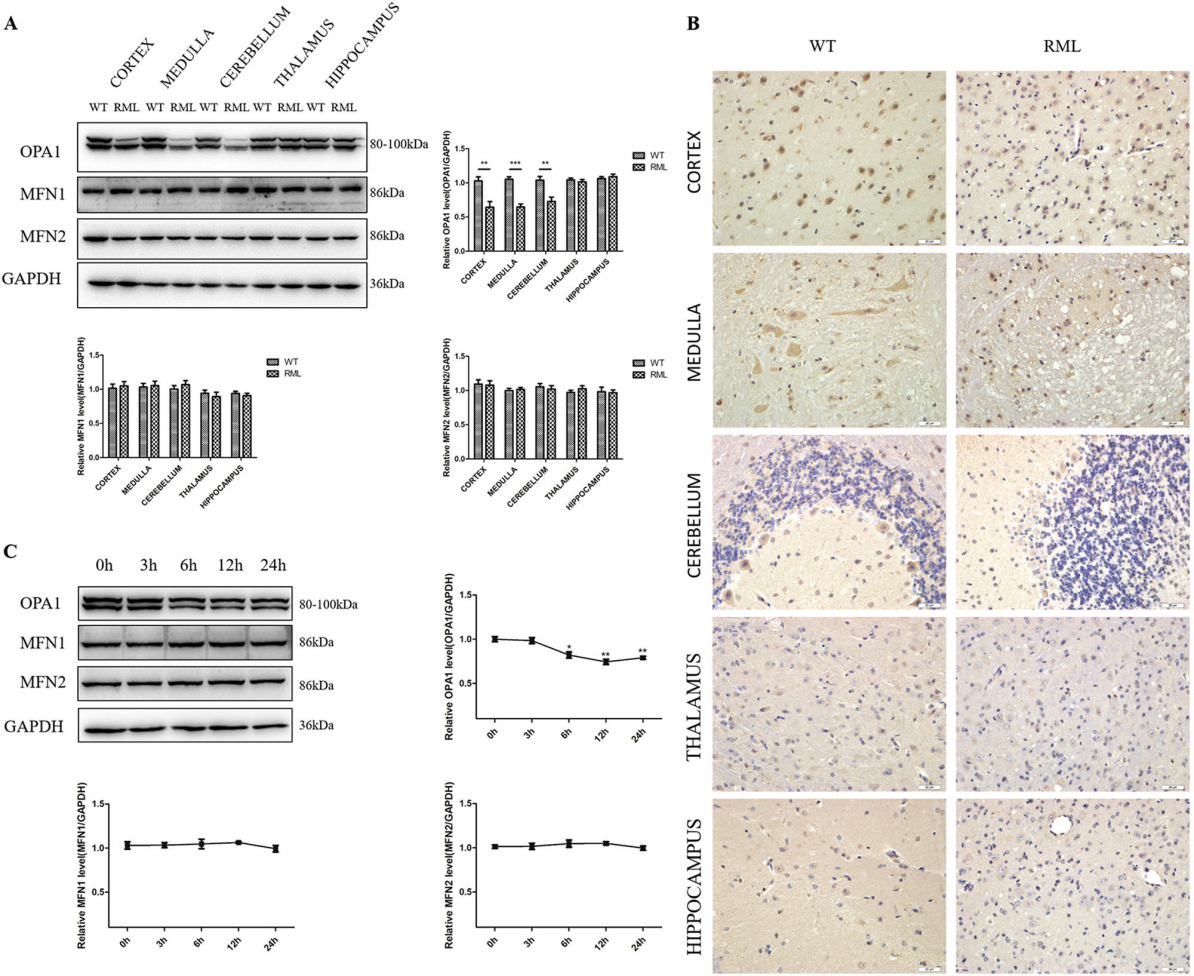
OPA1 is downregulated in prion disease models. **a** Representative western blots and quantitative results of OPA1, MFN1, and MFN2 in the brain of RML-infected C57 mice compared with the age-matched WT mice. **b** Immunohistochemistry (IHC) staining of OPA1 in the brain tissue of RML-infected C57 mice compared with the age-matched WT mice. **c** Representative western blots and quantitative results of OPA1, MFN1, and MFN2 in primary neuron cultures incubated with PrP^106–126^ (150 μM). Ten RML-infected C57 mice and ten age-matched WT mice were analyzed. All in vitro experiments were repeated at least three times. Data are mean ± SEM. (*P < 0.05, **P < 0.01, ***P < 0.001)

We previously reported abnormalities of mitochondrial morphology and function in prion diseases like other neurodegenerative diseases and increased mitochondrial DLP1 in both in vitro and in vivo prion models^23^. To search for other mitochondrial fusion proteins involved in mitochondrial network fragmentation and mitochondrial dysfunction in prion diseases, in this study we investigated the expression of mitochondrial fusion-regulating proteins, OPA1, MFN1, and MFN2. We infected C57 mice by intracerebral injection of the RML scrapie agent and studied RML-infected brain tissues at the terminal stage of prion disease. Western blotting results showed that the OPA1 protein in cerebral cortex, medulla, and cerebellum of RML-infected mice was markedly reduced compared with age-matched wild-type (WT) mice, while OPA1 protein levels in the thalamus and hippocampus were comparable between the RML-infected and WT mice (Fig. 1a). Brain MFN1 and MFN2 protein levels in the five regions were unaffected by prion infection (Fig. 1a). Then we further observed the distribution of OPA1 in the brain by IHC. Compared with the number of large and deep-stained cells and stain intensity in the age-matched WT mice, OPA1-specific signals in the cortex, medulla, and cerebellum regions of the RML-infected mice were markedly smaller and weaker and were associated with most serious lesions in these regions of the brain (plaques or vacuoles) but not in the thalamus and hippocampus (Fig. 1b).

In the in vitro prion model, the level of OPA1 in PrP^106–126^-treated primary neurons started to decrease at 6 h and remained low for 24 h, while the levels of MFN1 and MFN2 showed no significant difference between the infected and control cultures during the 24-h observation period (Fig. 1c). Taken together, OPA1 is downregulated in prion disease models in vivo and in vitro.

### OPA1 participates in regulation of mitochondrial network morphology in in vitro models of prion diseases

**Fig. 2:**
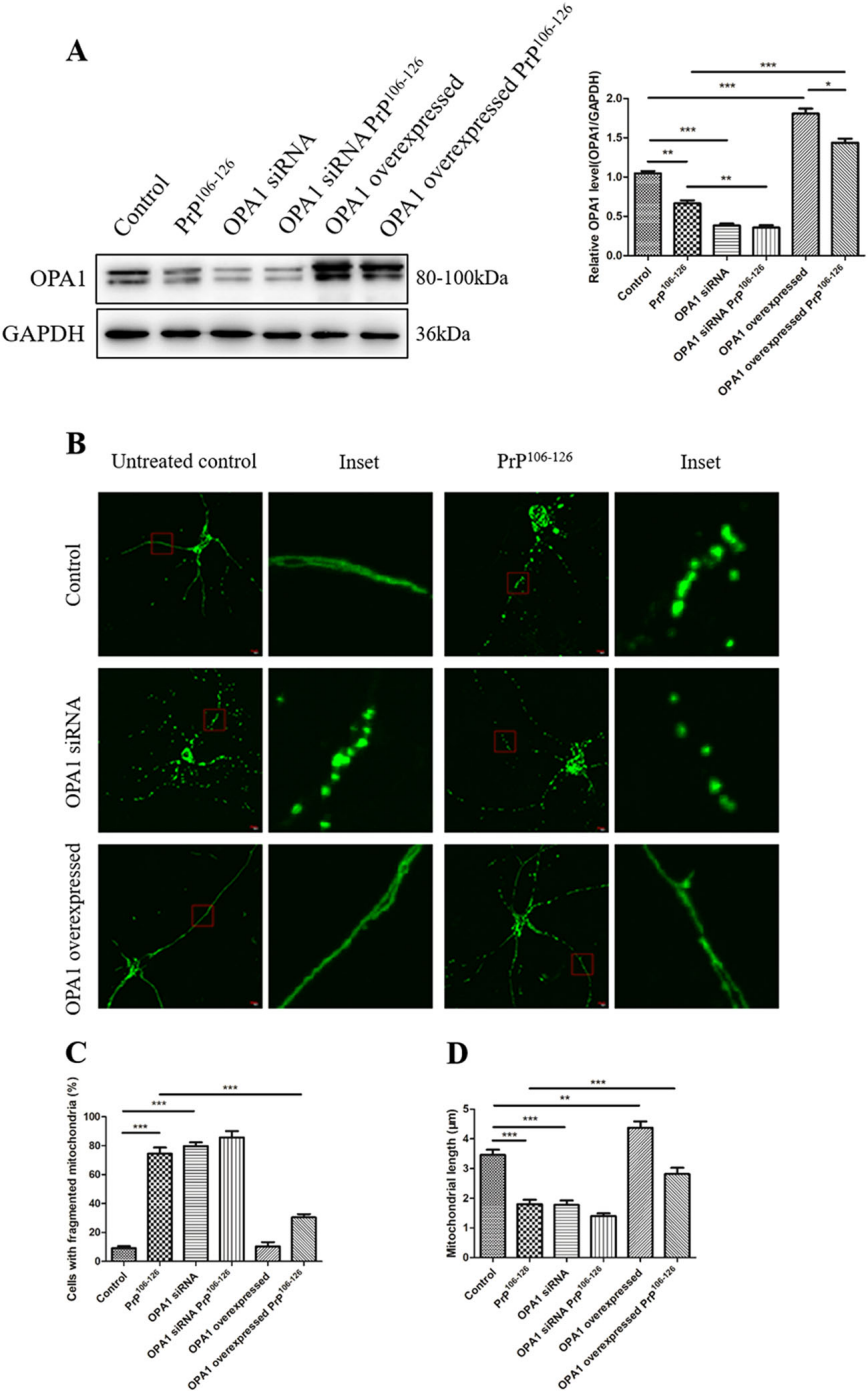
OPA1 participates in regulation of mitochondrial network morphology in in vitro models of prion diseases. **a** Representative western blots and quantitative results of OPA1 protein levels. Control, OPA1 siRNA, and OPA1-overexpressed primary neurons before and after incubation with PrP^106–126^ (150 μM) for 12 h. **b** Primary neurons were transfected with OPA1 siRNA or OPA1 overexpression plasmid together with mito-GFP and then incubated with PrP^106–126^ (150 μM) for 12 h. Mitochondria morphology was observed by confocal microscopy. **c** The percentage of cells with fragmented mitochondria. **d** Mitochondria length. All in vitro experiments were repeated at least three times. Data are means ± SEM. (*P < 0.05, **P < 0.01, ***P < 0.001)

The mitochondrial morphology changes through fusion and fission, which are regulated by mitochondrial fusion/fission proteins including OPA1 and DRP1. We next asked whether OPA1 mediates PrP^106–126^-induced abnormal mitochondrial morphology. We observed mitochondria morphology of neuron cells with suppressed or overexpressed OPA1 and PrP^106–126^ treatment. First, we transiently transfected neuron cells with OPA1 siRNA and OPA1 overexpression plasmid and determined the OPA1 protein level by western blotting 48 h after transfection. The level of OPA1 in OPA1-silenced neurons was reduced by about 65% and in OPA1-overexpressed neurons it was increased by about 1.8-fold (Fig. 2a), compared with untransfected controls.

Next, we expressed the primary neurons with mito-GPF, a green fluorescent protein that specifically targets the mitochondrial matrix, for the observation of mitochondria morphology. At 48 h, the primary neurons were incubated with 150 μM PrP^106–126^ for 12 h and then examined by fluorescence microscopy. PrP^106–126^-treated primary neurons showed severely fragmented network with small and round mitochondria (Fig. 2b), in comparison to untreated control neurons with the classical mitochondrial network of filamentous and tubular mitochondria. The percentage of PrP^106–126^-treated neurons with fragmented mitochondria was significantly increased to about 75% in comparison to <10% in untreated control neurons (Fig. 2c). Furthermore, compared to untreated control neurons, the length of mitochondria was significantly decreased in PrP^106–126^-treated neurons (Fig. 2d).

To further investigate the role of OPA1 in PrP^106–126^-induced mitochondrial network fragmentation, primary neurons were transiently transfected with OPA1 siRNA or OPA1 overexpression plasmid, both with mito-GFP. In OPA1 siRNA-transfected neurons, most cells showed fragmented network with short and round mitochondria and the length of mitochondria was significantly decreased compared with untransfected, untreated control neurons, similar to PrP^106–126^-treated neurons (Fig. 2b–d). OPA1 overexpression did not alter mitochondria network morphology but significantly inhibited PrP^106–126^-induced mitochondrial network fragmentation and significantly increased the length of mitochondria, showing <30% short and round mitochondria compared with PrP^106–126^-treated, untransfected neurons (Fig. 2b–d). Thus these results revealed that OPA1 is an important factor in PrP^106–126^-induced mitochondrial network fragmentation and preserves mitochondria morphology. Silencing of OPA1 aggravates mitochondrial network fragmentation, whereas overexpression of OPA1 rescues mitochondrial network morphology in the in vitro model of prion diseases.

### OPA1 controls mitochondrial cristae remodeling in prion disease models

**Fig. 3:**
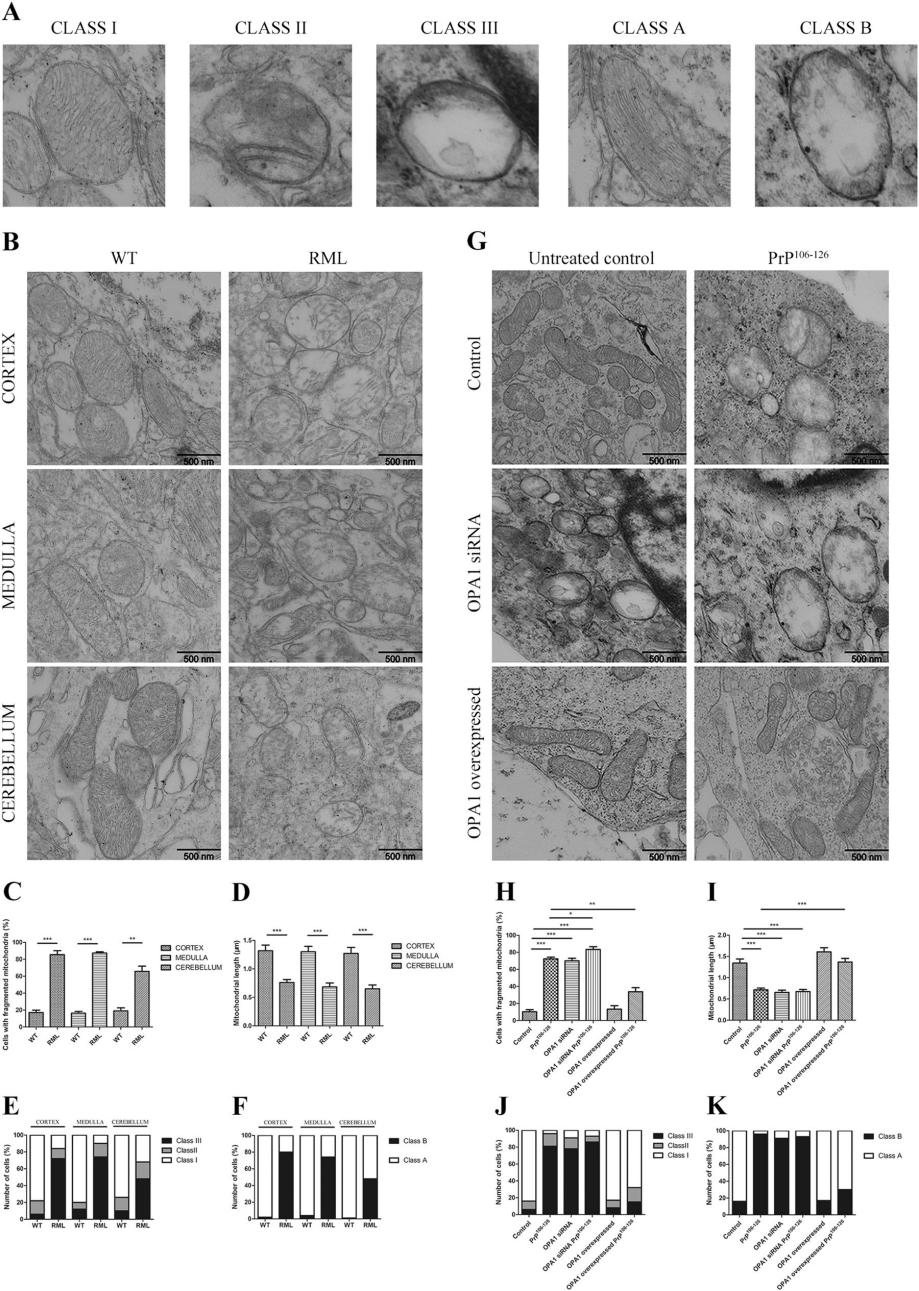
OPA1 controls mitochondrial cristae remodeling in prion disease models. **a** Representative electron microscopy (EM) images of mitochondrial cristae number and matrix density and swelling. **b** TEM images showing mitochondrial cristae remodeling in cortex, medulla, and cerebellum of RML-infected C57 mice. **c** The percentage of cells with fragmented mitochondria. **d** Mitochondria length. **e** Fifty mitochondria per sample were scored in three categories: more than four cristae (Class I), between two and three cristae (Class II), and one or no cristae (Class III) per mitochondrion. **f** Fifty mitochondria per sample were scored according to matrix density and swelling: dense matrix (Class A) and swollen mitochondria with hypodense matrix (Class B). **g** TEM images showing mitochondrial cristae remodeling in OPA1 siRNA- and OPA1-overexpressed primary neurons incubated with PrP^106–126^ (150 μM) for 12 h. **h** The percentage of cells with fragmented mitochondria. **i** Mitochondria length. **j** Fifty mitochondria per sample were scored in three categories: more than four cristae (Class I), between two and three cristae (Class II), and one or no cristae (Class III) per mitochondrion. **k** Fifty mitochondria per sample were scored according to matrix density and swelling: dense matrix (Class A) and swollen mitochondria with hypodense matrix (Class B). Ten RML-infected C57 mice and ten age-matched WT mice were analyzed. All in vitro experiments were repeated at least three times. Data are means ± SEM. (*P < 0.05, **P < 0.01, ***P < 0.001)

Next, we asked whether OPA1 mediates PrP^106–126^-induced abnormal cristae ultrastructure. Mitochondrial ultrastructure in prion disease models in vivo and in vitro were observed by electron microscopy. Mitochondria were classified by the number of cristae or swelling into the following categories based on the number of cristae or mitochondria swelling: Class I (more than four cristae), Class II (two to three cristae), Class III (no or one cristae), Class A (dense matrix), and Class B (swollen mitochondria with hypodense matrix) according to Del Dotto et al. (Fig. 3a)^24^. In WT mice, brain cortex, medulla, and cerebellum had approximately 80% of mitochondria belonging to Class I and >95% of Class A (Fig. 3b–f). On the contrary, in RML-infected mice, mitochondria of these regions of brain displayed abnormal ultrastructure, with approximately 60–80% damaged mitochondria characterized as markedly shorter mitochondria, reduced cristae number, and swelling (Fig. 3b–f). These findings indicated mitochondrial cristae remodeling in the in vivo prion disease model.

Next, we observed mitochondrial ultrastructure in PrP^106–126^-treated primary neurons. Consistent with the in vivo results, the prion peptide severely damaged mitochondria, with decreased mitochondrial length and increased fragmented mitochondria with about 81% Class III and 95% Class B mitochondria (Fig. 3g–k). We also observed that mitochondrial ultrastructure of OPA1-silenced neurons was similar to the prion peptide-treated cells, showing only 9% Class I and Class A mitochondria (Fig. 3g–k). Damaged mitochondrial ultrastructure in PrP^106–126^-treated primary neurons, the percentage of fragmented mitochondria, and the length of mitochondria were markedly alleviated by overexpression of OPA1, which rescued mitochondrial cristae to 68% Class I and 15% Class III and the percentage of Class A mitochondria to 70% in the in vitro prion model (Fig. 3g–k). Thus we demonstrated that OPA1 is an important factor in PrP^106–126^-induced mitochondrial cristae remodeling. Silencing OPA1 aggravates mitochondrial cristae damage, and overexpression of OPA1 alleviates cristae ultrastructure damage in the in vitro prion model.

### Overexpression of OPA1 ameliorates PrP^106–126^-induced mitochondrial dysfunction

We further determined whether OPA1 could affect PrP^106–126^-induced change of mitochondrial function in the in vitro prion model. Maintaining a normal ADP/ATP ratio and ATP level is essential for cell and tissue structure and function.

**Fig. 4:**
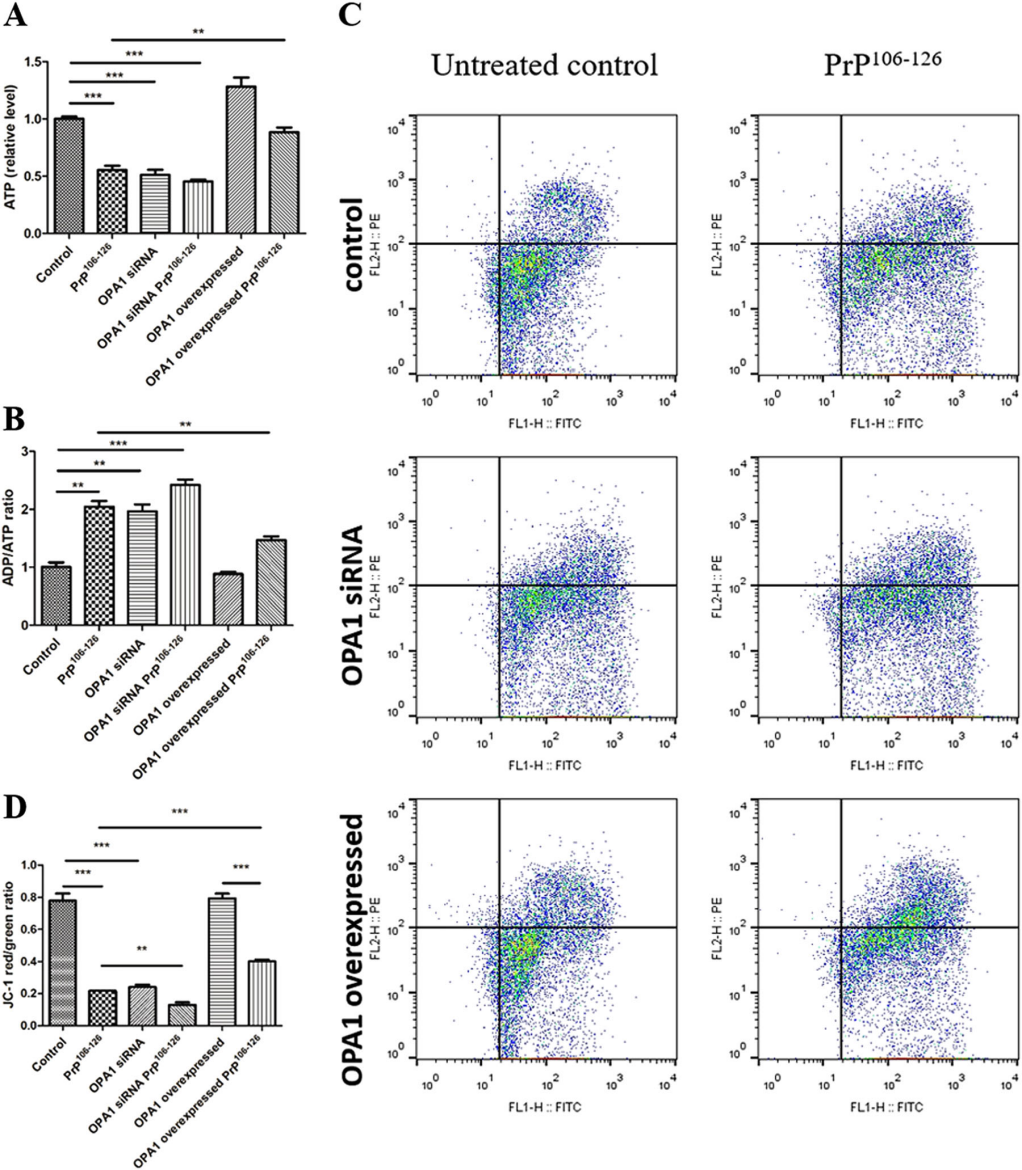
Overexpression of OPA1 ameliorates PrP^106–126^-induced mitochondrial dysfunction. Control, OPA1-silenced, and OPA1-overexpressed primary neurons were incubated with PrP106-126 (150 μM) for 12 h. Intracellular ATP levels (**a**), ADP/ATP ratio (**b**), and mitochondrial membrane potential (**c**, **d**) were determined. Mitochondrial membrane potential is expressed as JC-1 monomer (green fluorescence)/JC-1 aggregates (red fluorescence) ratio. All in vitro experiments were repeated at least three times. Data are means ± SEM. (**P < 0.01, ***P < 0.001)

Mitochondrial function was assessed after incubation with 150 μM PrP^106–126^ for 12 h. We found that the intracellular ATP level was decreased to 50% and the ADP/ATP ratio increased by twofold, relative to the untreated control neurons (Fig. 4a, b). Similar results were observed in OPA1-suppressed neurons (Fig. 4a, b). However, OPA1 overexpression prevented prion-induced ATP loss and the increase in ADP/ATP ratio (Fig. 4a, b).

Mitochondrial membrane potential (MMP) is also a key maker of mitochondrial function. Thus we assessed mitochondrial function by the JC-1 MMP assay. In concert with the above findings, after treatment with PrP^106–126^, OPA1 siRNA-transfected neurons displayed increased green fluorescence (JC-1 monomer form) and decreased red fluorescence (JC-1 aggregate form) (Fig. 4c, d), indicating low MMP values and thus mitochondrial dysfunction compared with untreated control neurons. OPA1 knockdown significantly aggravated MMP alteration by the prion peptide, whereas the overexpression of OPA1 alleviated the effect of the prion peptide on MMP (Fig. 4c, d).

In summary, these results indicated that OPA1 is vital in maintaining mitochondrial function; OPA1 overexpression alleviated PrP^106–126^-induced mitochondrial dysfunction.

### Overexpression of OPA1 alleviates PrP^106–126^-induced mtDNA depletion

**Fig. 5:**
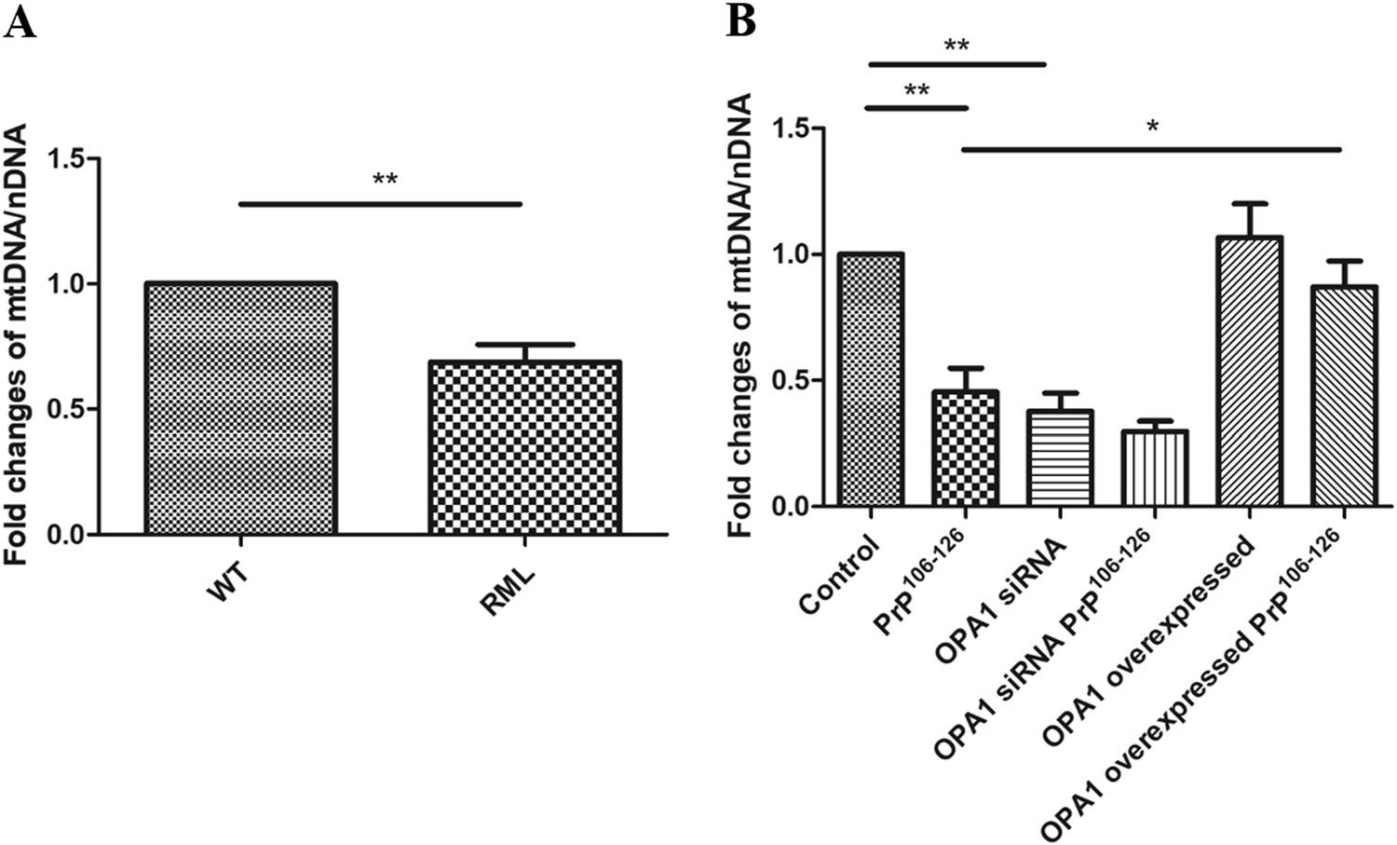
Overexpression of OPA1 alleviates PrP^106–126^-induced mtDNA depletion. **a** Relative mitochondrial copy number (mtDNA/nDNA) in RML-infected C57 mice brains compared with the age-matched control. **b** Relative mitochondrial copy number (mtDNA/nDNA) in OPA1 siRNA-transfected and OPA1-overexpressed primary neurons incubated with PrP^106–126^ (150 μM) for 12 h. Ten RML-infected C57 mice and ten age-matched control mice were analyzed. All in vitro experiments were repeated at least three times. Data are means ± SEM. (*P < 0.05, **P < 0.01)

To further explore the effects of prion infection on mtDNA, we measured mtDNA number. It has been previously reported that the ratio of mtDNA to nDNA represents relative mitochondria content^25,26^. The mtDNA copy number in RML mice brain decreased by approximately 40%, compared to WT mice brain (Fig. 5a). Similarly, we observed that PrP^106–126^-treated primary neurons contain about 50% of mtDNA copy number of untreated control neurons (Fig. 5b). Concordantly, similar data were found in OPA1 siRNA-transfected neurons, which had about 40% mtDNA of untreated control neurons, suggesting that OPA1 is a crucial factor in maintaining mtDNA (Fig. 5b). We further investigated whether overexpression of OPA1 can alleviate mtDNA loss in PrP^106–126^-treated primary neurons (Fig. 5b). OPA1 overexpression blocked PrP^106–126^-induced mtDNA loss; the mtDNA/nDNA ratio in OPA1-overexpressed primary neurons was 80% of that in the control neurons, compared with twofold in the prion-treated WT neurons. In summary, these results indicate that OPA1 is a crucial factor in mtDNA depletion in prion diseases. OPA1 overexpression alleviated PrP^106–126^-induced mtDNA depletion.

### OPA1 overexpression protects from PrP^106–126^-induced neuron apoptosis

**Fig. 6:**
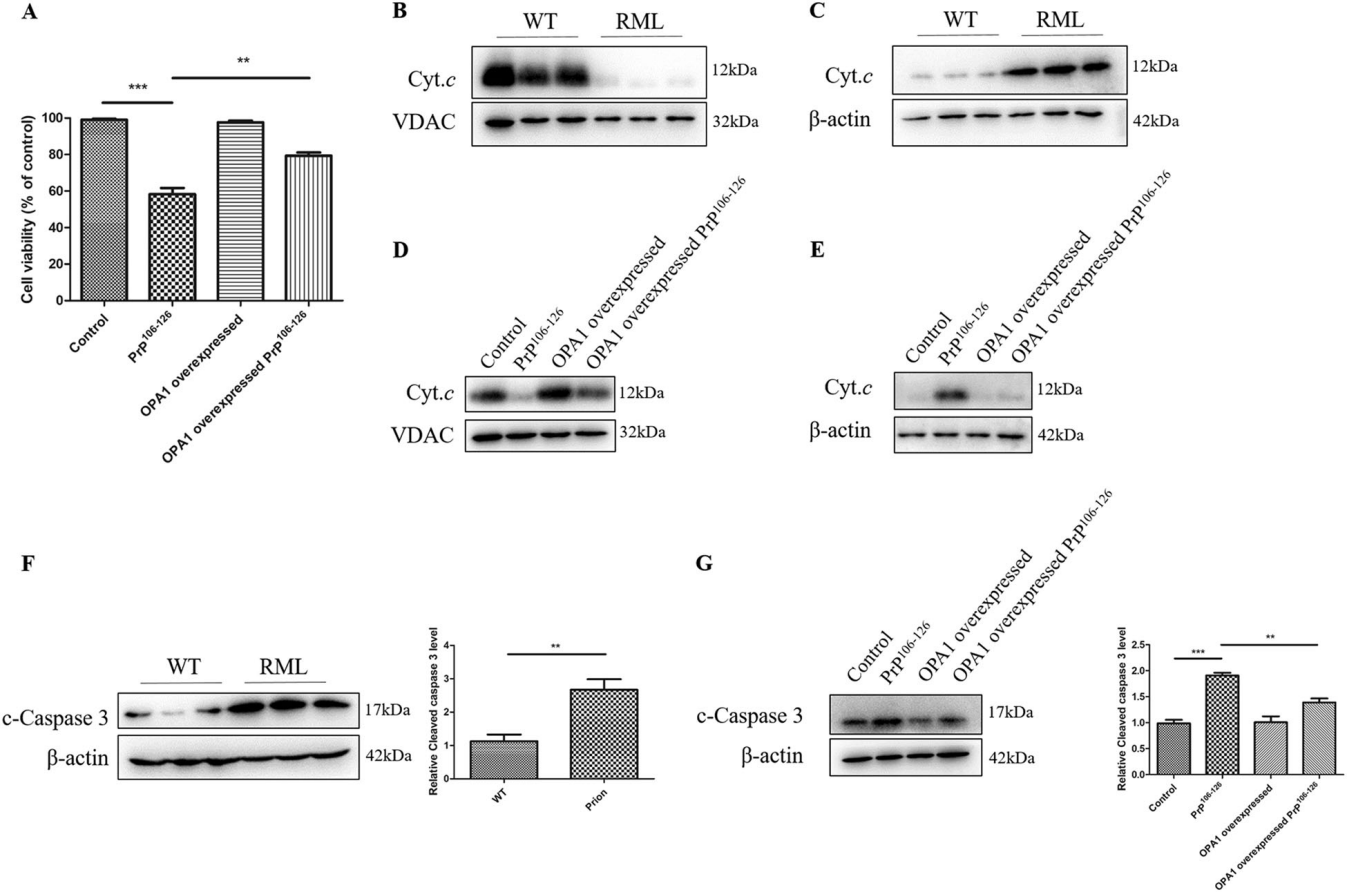
OPA1 overexpression protects from PrP^106–126^-induced neuron apoptosis. **a** Control and OPA1-overexpressed primary neurons were incubated with PrP^106–126^ (150 μM) for 24 h, and cell viability was measured by the CCK-8 Kit. **b** Immunoblots of mitochondrial cytochrome c proteins extracted from RML-infected C57 mouse brains, compared with the age-matched control. **c** Immunoblots of cytosol cytochrome c extracted from RML-infected C57 mouse brains, compared with the age-matched control. **d** Immunoblots of mitochondrial cytochrome c proteins of primary neurons. **e** Immunoblots of cytosol cytochrome c of primary neurons. **f** Western blots and quantitative results of cleaved caspase 3 levels in RML-infected C57 mouse brains compared with the age-matched control. **g** Western blots and quantitative results of cleaved caspase 3 levels in primary neurons. Ten prion-infected mice and wild-type mice were analyzed. All in vitro experiments were repeated at least three times. Data are means ± SEM. (**P < 0.01, ***P < 0.001)

The accumulation of mitochondrial cristae remodeling and mitochondrial dysfunction results in cell death. As one of the cellular functions of OPA1 was preventing apoptosis by controlling the release of cytochrome c^20^, we further tested the role of OPA1 in PrP^106–126^-induced cell death. First, we measured the cell viability by the CCK-8 assay in PrP^106–126^-treated primary neurons. Compared with the untreated control neurons, the cell viability of PrP^106–126^-treated primary neurons decreased to about 60% (Fig. 6a). However, the percentage of surviving neurons relative to PrP^106–126^-treated primary neurons significantly increased from 60% to 80% in neurons with overexpressed OPA1 (Fig. 6a).

It is well known that cytochrome c proteins act downstream in the mitochondrial apoptotic pathway. Then, to further confirm the function of OPA1 in PrP^106–126^-induced cell death, we overexpressed OPA1 protein in primary neurons, treated with 150 μM PrP^106–126^ for 24 h. Primary neurons and RML-infected mouse brains were fractionated to mitochondrial and cytosolic extracts and mitochondrial and cytosol cytochrome c protein levels were measured by western blot analysis (Fig. 6b–e). We found that mitochondrial cytochrome c was severely decreased and cytosol cytochrome c was severely increased in RML-infected mouse brain, suggesting release of cytochrome c, compared with the WT mouse brain (Fig. 6b, c). Consistent with the in vivo results, cytochrome c release also occurred in PrP^106–126^-treated primary neurons (Fig. 6e, f). Overexpression of OPA1 significantly inhibited the release of cytochrome c (Fig. 6e, f).

Activation of caspase 3 is important in apoptotic cell death in many chronic neurodegenerative diseases. We studied whether overexpression of OPA1 repressed the activation of caspase 3. The levels of cleaved caspase 3 in the brains of terminal-stage RML-infected mice was significantly increased, compared with WT mice (Fig. 6f). Similarly, prion peptide treatment of neuronal cells also led to caspase 3 activation and cell death (Fig. 6a, g). Overexpression of OPA1 significantly reduced caspase 3 activation induced by PrP^106–126^ (Fig. 6g).

In summary, these results indicated that OPA1 overexpression prevented PrP^106–126^-induced neuron apoptosis.

## Discussion

Prion diseases are a family of fatal neurodegenerative disorders, with long incubation periods and characteristic spongiform changes in the brain associated with the progressive loss of neuronal cells and their function. The mechanisms of neurodegeneration remain unclear and effective therapeutic targets have not been discovered despite decades of research by scientists worldwide. In this study, we found that OPA1 protein, which regulates IMM events, is downregulated in in vitro and in vivo prion disease models, and overexpression of OPA1 blocked prion-induced mitochondrial network fragmentation and mitochondrial dysfunction, loss of mtDNA, and neuronal apoptosis, suggesting that OPA1 could be an effective therapeutic target for the treatment of early-stage prion diseases.

We demonstrated that OPA1 expression significantly decreased in cerebral cortex, medulla, and cerebellum of RML-infected mice and PrP^106–126^-treated primary neurons, similar to the findings in the brains of 139A-, ME7-, 263K- and S15-infected mice^22^. In addition, downregulated OPA1 has been reported in other neurodegenerative diseases models, such as Alzheimer’s disease, Parkinson’s disease, Huntington’s disease (HD), and retinal ischemia–reperfusion-related diseases^27–31^. Mitochondria with suppressed OPA1 were incapable of fusion^32,33^. OPA1-knockdown mouse embryonic fibroblasts exhibited mitochondrial network fragmentation, abnormal ultrastructure, less mitochondrial cristae number, mitochondrial swelling, and decreased mtDNA copy numbers^24,26,34^. Re-expression of any OPA1 isoform ameliorated cristae abnormality, mtDNA abundance, and energetic efficiency. Similarly, we showed that overexpression of OPA1 prevented mitochondrial network fragmentation, cristae remodeling, and mtDNA depletion in PrP^106–126^-treated primary neurons.

Maintaining normal ATP level and MMP is critical for neuronal survival. In neurodegenerative diseases, mitochondrial network fragmentation and disruption of cristae structure, as a result of reduced ATP levels and dissipated MMP, are responsible for neuronal dysfunction^23,35,36^. Our observation of marked reduction in ATP and MMP loss in the in vitro prion model suggests neuronal dysfunction. Depletion of OPA1 resulted in ATP and MMP loss and induced OPA1-depentent mitochondrial fragmentation and cristae damage. Not surprisingly, we found that overexpression of OPA1 abolished the effects of prion on the ATP level and MMP, as well as mitochondrial structure damage by the prion peptide. In addition, it was reported that overexpression of OPA1 improved respiratory chain efficiency in two mouse models of oxidative phosphorylation complex I and IV deficiency^37^. We will further study the impact of OPA1 overexpression on respiratory chain in prion disease models.

It has been documented that increases of several apoptosis factors including cleaved caspase 3 have been found in the brain tissues of hamsters infected with 263K^23^ and mice infected with 139A or ME7^22^. We also found a significant increase of cleaved caspase 3 and release of cytochrome c in RML-infected mice brain tissues and an increase of cleaved caspase 3, release of cytochrome c, and a decline of cell viability in PrP^106–126^-treated primary neurons, suggesting cell apoptosis in prion diseases. In our in vitro prion model, overexpression of OPA1 prevented release of mitochondrial cytochrome c and activation of caspase 3 and increased cell viability. It has been documented that silence of OPA1 caused mitochondrial fragmentation and severe cristae remodeling and heightened release of cytochrome c and activation of caspase-dependent apoptosis in HeLa cells^20^. Intriguingly, it was reported that OPA1-dependent mitochondrial cristae remodeling controls apoptotic independently from mitochondrial fusion, and OPA1 protects cells from apoptosis by maintaining cristae junctions tight and cristae density and controlling the release of cytochrome c^19^. Mild overexpression of OPA1 prevented muscular atrophy, heart and brain ischemia, and liver apoptosis by OPA1-dependent mitochondrial cristae remodeling^38^. In a mouse HD model, overexpression of OPA1, but not the overexpression of the fusion factor MFN1, reversed the increased susceptibility to cell death inherent in the HD model, suggesting that enhanced fusion alone is not sufficient to inhibit apoptosis, further highlighting the crucial role of OPA1-controlled cristae remodeling in apoptosis^31^. Furthermore, mitochondrial fragmentation may be essential for cytochrome c redistribution^39,40^ and mitochondrial dynamics, and the delicate balance between fusion and fission is very important in the timely execution of apoptosis^9,41^. Inhibition of DRP1-mediated fission or excess mitochondrial fusion induced by overexpression of OPA1, MFN1, and/or MFN2 diminishes or delays apoptotic stimuli-induced mitochondrial fragmentation, cytochrome c release, and cell death, whereas overexpression of DRP1 or downregulation of OPA1, MFN1, or MFN2 aggravates apoptosis^42^. Published and our current study results support the notion that OPA1 inhibits apoptosis through enhancing mitochondria fusion and maintaining mitochondria cristae integrity.

Our work indicates that overexpression of OPA1 alleviates mitochondrial damage in prion diseases by preventing mitochondrial network fragmentation, disorganization of cristae structure, mtDNA loss, mitochondrial dysfunction, and cell death. OPA1 is a potential therapeutic target in prion diseases.
